# Apically localized PANX1 impacts neuroepithelial expansion in human cerebral organoids

**DOI:** 10.1038/s41420-023-01774-7

**Published:** 2024-01-11

**Authors:** Rebecca J. Noort, Hanrui Zhu, Robert T. Flemmer, Craig S. Moore, Thomas J. Belbin, Jessica L. Esseltine

**Affiliations:** 1 Division of BioMedical Sciences, Faculty of Medicine, Memorial University of Newfoundland, St. John’s, A1B 3V6 NL Canada; 2 Discipline of Oncology, Faculty of sp. Medicine, Memorial University of Newfoundland, St. John’s, A1B 3V6 NL Canada

**Keywords:** Cell adhesion, Neurogenesis

## Abstract

Dysfunctional paracrine signaling through Pannexin 1 (PANX1) channels is linked to several adult neurological pathologies and emerging evidence suggests that PANX1 plays an important role in human brain development. It remains unclear how early PANX1 influences brain development, or how loss of PANX1 alters the developing human brain. Using a cerebral organoid model of early human brain development, we find that PANX1 is expressed at all stages of organoid development from neural induction through to neuroepithelial expansion and maturation. Interestingly, PANX1 cellular distribution and subcellular localization changes dramatically throughout cerebral organoid development. During neural induction, PANX1 becomes concentrated at the apical membrane domain of neural rosettes where it co-localizes with several apical membrane adhesion molecules. During neuroepithelial expansion, *PANX1*−/− organoids are significantly smaller than control and exhibit significant gene expression changes related to cell adhesion, WNT signaling and non-coding RNAs. As cerebral organoids mature, PANX1 expression is significantly upregulated and is primarily localized to neuronal populations outside of the ventricular-like zones. Ultimately, PANX1 protein can be detected in all layers of a 21–22 post conception week human fetal cerebral cortex. Together, these results show that PANX1 is dynamically expressed by numerous cell types throughout embryonic and early fetal stages of human corticogenesis and loss of PANX1 compromises neuroepithelial expansion due to dysregulation of cell-cell and cell-matrix adhesion, perturbed intracellular signaling, and changes to gene regulation.

## Introduction

Human brain development follows a series of intricately choreographed events involving large cellular migrations and rearrangements, changes in cell morphology, and cell fate specification. These activities are locally organized through exquisite spatial and temporal control of signaling events between neighboring cells. Dysfunctional paracrine signaling through Pannexin 1 (PANX1) channels is linked to several adult neurological pathologies and human germline *PANX1* variants have been associated with severe neurological deficits and autism spectrum disorder [1, 2]. Studies in postnatal rodent models reveal PANX1 expression across various neural cell types including neurons, glia, and neural progenitor cells [3–5]. In postnatal murine neural precursor cells (NPCs), PANX1 restricts neuronal differentiation by impeding neurite extension and cell migration via the channels’ ATP release functions and interactions with the cytoskeleton [6, 7]. Others have demonstrated PANX1 localization at neuronal synapses where the channels help to replenish extracellular ATP, negatively regulate dendritic spine density, and maintain synaptic strength [8, 9]. However, it remains unclear how early PANX1 influences human brain development, or which cell types express PANX1 in the developing human brain.

Recent reports have revealed that PANX1 is expressed in some of the earliest cell types in human development including human oocytes, pluripotent stem cells, and the three embryonic germ layers (definitive endoderm, mesoderm, and ectoderm) [10–12]. PANX1 channels are also expressed throughout embryonic brain development. *PANX1* transcript expression is robust in the developing mouse cerebral cortex, cerebellum, and olfactory bulbs where maximum *PANX1* expression occurs at murine embryonic day 18 and declines thereafter [13]. Gene expression analyses curated by BrainSpan indicate that a similar pattern occurs in the human system as *PANX1* transcript expression in various brain structures is high at 8 post conception weeks (pcw) (earliest timepoint assessed) but diminishes around 26 pcw (Brainspan.org). The Human Protein Atlas reports moderate-to-high PANX1 protein abundance in the adult human cerebral cortex (humanproteinatlas.org). Given this dynamic pattern of PANX1 expression, we expect that PANX1-mediated cellular communication influences proper development of neural tissues.

To date, the cellular and subcellular localization of PANX1 protein throughout human embryonic and early fetal brain development have not been investigated. Here we use iPSC-derived neural precursor cells, neurons, and cerebral organoids to investigate PANX1 expression and localization as iPSCs differentiate to neural cell types and organized cortical structures. Cerebral organoids recapitulate a variety of human brain regions including the cerebral cortex, hippocampus, choroid plexus, and retinal tissue and contain a variety of cell types including neural progenitors (like neuroepithelial cells and radial glia), neurons, astrocytes, oligodendrocytes, retinal pigment epithelial cells, and ependymal cells [14–17]. Importantly, the cells within cerebral organoids self-organize to form cortical-like layers like those seen in the developing human brain [14] making cerebral organoids a powerful tool to study the embryonic and early fetal stages of human brain development.

Given that PANX1 is expressed in the earliest cell types of human development and is linked to neurological disease, we sought to explore PANX1 expression and localization throughout early stages of human brain development. Immunostaining of a 21–22 pcw (midgestation) human fetal cerebral cortex reveals PANX1 protein expression in all cortical layers, with heightened signal intensity in the marginal zone. We observe concentrated PANX1 expression at the apical membrane domain of neuroepithelial-stage iPSC-derived cerebral organoids whereas more mature organoids exhibit the heaviest PANX1 expression within the emerging neuronal layers. CRISPR-Cas9 *PANX1* gene ablation results in stunted neuroepithelial expansion and dysregulation of genes related to cell signaling, cell adhesion, and expression of non-coding RNAs.

## Results

### PANX1 is expressed across the human fetal cerebral cortex

**Fig. 1:**
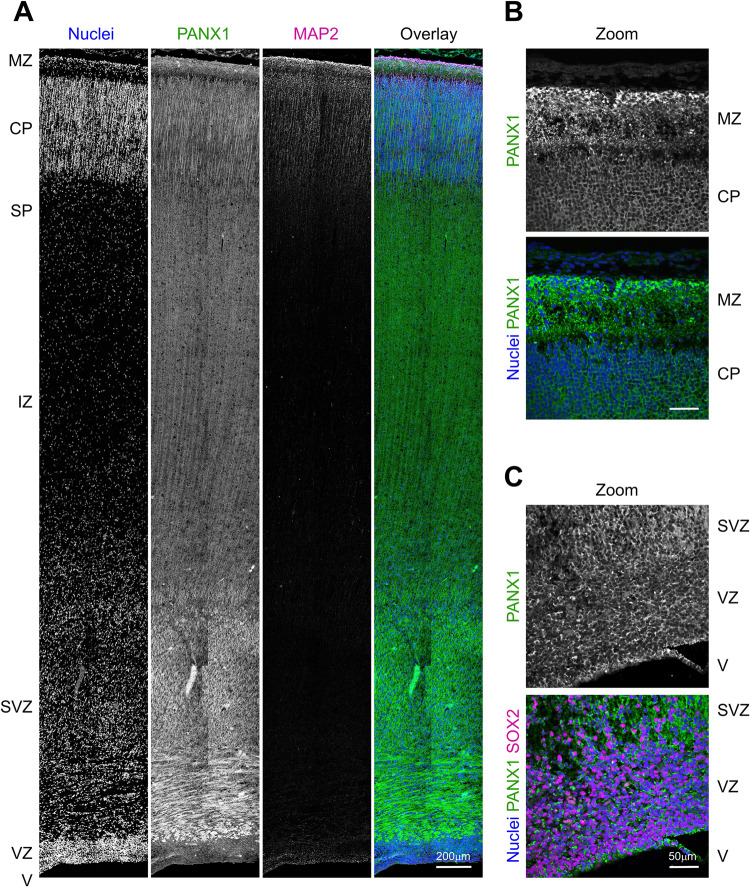
PANX1 protein is expressed across all layers of the human fetal cerebral cortex. **A** Representative immunofluorescence confocal micrograph of PANX1 (green) along with MAP2 (magenta) across the span of a 21–22 week (midgestation) human fetal cerebral cortex. Higher resolution confocal micrographs demonstrating PANX1 cellular distribution in **B** the outer cortical layers and **C** the ventricular zone. Nuclei (Hoechst, blue). Scale bars as indicated. MZ marginal zone, CP cortical plate, SP subplate, IZ intermediate zone, SVZ subventricular zone, VZ ventricular zone, V ventricle.

The PANX1 literature heavily favors perinatal or postnatal mouse systems. However, the Allen Institute’s Brainspan prenatal laser microdissection (LMD) microarray dataset depicts *PANX1* transcript expression in 21 pcw human fetal brains including cortical regions such as the ventricular zone (VZ), subventricular zone (SVZ), intermediate zone (IZ), subplate (SP), cortical plate (CP), and marginal zone (MZ) (Brainspan.org). To confirm whether PANX1 protein is also expressed in these developing human tissue layers, we performed immunofluorescence confocal imaging on cortical samples from a 21–22 pcw human fetal brain (Fig. 1). We find PANX1 signal across all layers of the developing human cerebral cortex with widespread staining throughout the SVZ and brighter manifestation in the marginal zone (Fig. 1A). PANX1 signal intensity is diminished in regions with tightly packed nuclei, such as the cortical plate (Fig. 1A). In contrast, cortical layers with fewer nuclei such as the marginal zone and subplate display widespread PANX1 staining, concentrated throughout the many processes of MAP2-positive neurons (Fig. 1A, B). Interestingly, while we did see some evidence of PANX1 expression within the SOX2-positive stem cells lining the ventricular zone, this staining was much diminished compared to the more mature neuronal layers within the human fetal cortex (Fig. 1C). Collectively, we find that PANX1 protein expression is apparent in all cortical layers of the early fetal human brain.

### PANX1 is upregulated in neural progenitor cells and neurons compared to undifferentiated iPSCs

**Fig. 2:**
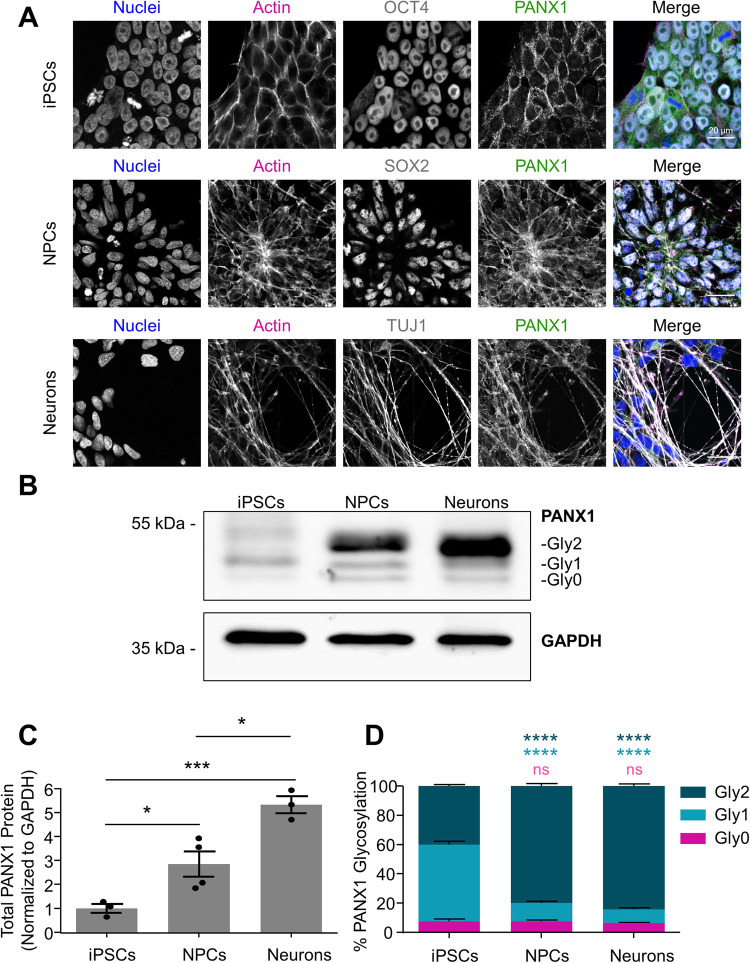
PANX1 protein expression is significantly upregulated following human iPSC differentiation to NPCs and neurons. **A** Representative immunofluorescence confocal micrographs depicting PANX1 protein (green) localization in OCT4-positive (gray) iPSCs, SOX2-positive (gray) NPCs, and TUJ1-positive (gray) neurons. Nuclei (Hoechst or To-Pro™−3 iodide, blue); Actin (phalloidin, magenta). Scale bar = 20 μm. **B** Representative Western blot depicts PANX1 protein expression as three discrete bands corresponding to the putative heavily glycosylated (Gly2), high mannose (Gly1), and non-glycosylated species (Gly0) in human iPSCs, NPCs, and neurons. **C** Densitometry analysis of total PANX1 protein expression in iPSCs, differentiated NPCs and differentiated neurons. Data normalized to GAPDH and expressed as a fold of undifferentiated iPSCs. **D** Densitometric analysis of the proportion of Gly2, Gly1 and Gly0 PANX1 species expressed as a percent of the total PANX1 protein present. Error bars depict the standard error of the mean where data is representative of 3–4 independent experiments. *p < 0.05; ***p < 0.001; ****p < 0.0001; ns nonsignificant relative to iPSCs.

The first step to determining how PANX1 influences human brain development is to uncover when and where PANX1 is expressed in the developing human brain. However, human fetal brain samples are precious and few, and a lot of development has already occurred even at the 21–22 pcw timepoint presented in Fig. 1. Therefore, once we confirmed PANX1 expression in a 21–22 pcw human fetal cortex, we evaluated PANX1 expression and localization in human iPSCs in vitro, and after differentiation into neural precursor cells (NPCs) and mature neurons. As we previously reported, PANX1 protein localized primarily to the cell periphery of undifferentiated iPSCs, where it colocalized with actin (Fig. 2A). PANX1 was similarly colocalized with actin in SOX2-expressing NPCs and TUJ1-expressing neurons (Fig. 2A). NPCs in culture typically form polarized neural rosettes, identified by Nestin/SOX2 expression and characteristic flower petal arrangement [18]. Interestingly, as the NPCs in culture arranged into neural rosette-like structures, we observed PANX1 staining concentrated at the centermost (apical) side of the neural rosettes (Fig. 2A). Western blotting revealed a significant upregulation of PANX1 protein as iPSCs differentiate toward NPCs and neurons (Fig. 2B, C). Indeed, NPCs express 2.852 ± 0.522-fold more PANX1 and neurons express 5.324 ± 0.357-fold more PANX1 compared to undifferentiated iPSCs (Fig. 2B, C). Additionally, we noted a difference in the PANX1 banding pattern on Western blots where NPCs and neurons possess a significantly greater proportion of the high molecular weight PANX1 isoform, most likely corresponding to the heavily glycosylated Gly2 species (Fig. 2B, D). The putative Gly2 PANX1 species comprises 40.150 ± 0.843% of total PANX1 in iPSCs, 79.848 ± 1.551% in NPCs, and 84.370 ± 1.357% in neurons (Fig. 2D). This dramatic upregulation of PANX1 protein during NPC and neuron differentiation suggests a role for PANX1 in neural specification and early human brain development.

### PANX1 is apically expressed in budding neuroepithelia of iPSC-derived cerebral organoids

**Fig. 3:**
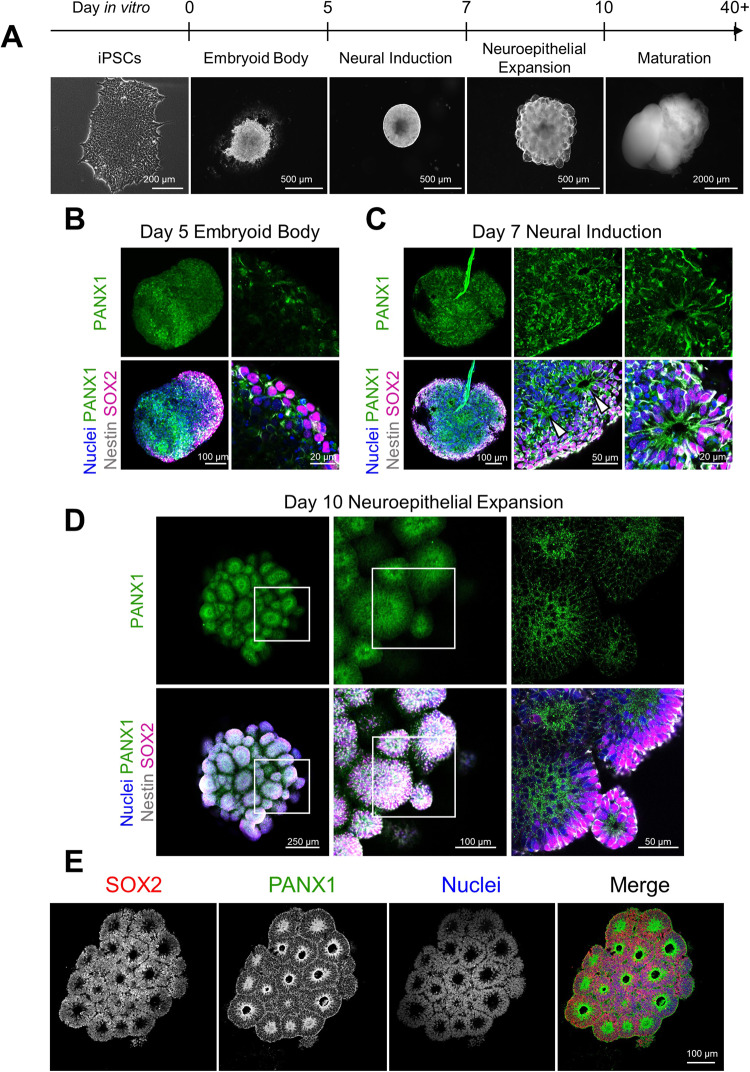
PANX1 is expressed at the earliest timepoints of cerebral organoid development. **A** Schematic depicting the various stages of cerebral organoid culture and representative phase images of organoid morphology. **B**–**D** Representative immunofluorescent confocal micrographs of whole-mount control cerebral organoids at various developmental stages labelled for PANX1 (green), Nestin (gray), and SOX2 (magenta). **B** PANX1 is widely expressed across the EB stage of cerebral organoid development (day 5). **C** At the end of the neural induction stage (day 7), PANX1 is beginning to be apically localized in SOX2-positive neural rosettes (white arrowheads). **D** Day 10 cerebral organoids at the end of the neuroepithelial expansion stage develop many bulbous regions of radially arranged neuroepithelia with PANX1 staining concentrated at the center. **E** Immunofluorescence confocal imaging of cryosectioned day 10 cerebral organoids demonstrating radially arranged SOX2-positive neuroepithelia (red) with PANX1 staining (green) concentrated at the center. Scale bars as indicated. Nuclei (Hoechst, blue).

To further understand how PANX1 influences the earliest stages of human brain development, we next employed a cerebral organoid model to evaluate PANX1 localization throughout the embryonic and early fetal stages of human cortex development. Cerebral organoids are generated through (1) 3D induction of neuroectoderm from stem cell-derived embryoid bodies (EBs); (2) arrangement of neural rosettes and neuroepithelial expansion; (3) ventricular-like zone formation and intermediate progenitor emergence; (4) neuronal differentiation and cortical layering (Fig. 3A). Ultimately, this results in a large, layered organoid comprised of numerous neural lineages.

At the end of the EB stage (day 5), organoids appear as a dense, disorganized cellular mass exhibiting some SOX2 and Nestin-positive regions. Whole-mount immunofluorescence confocal microscopy revealed wide PANX1 expression throughout the EB (Fig. 3B). Upon the initiation of neural induction (day 7), organoids begin to arrange into pseudostratified neural rosettes lined by polarized SOX2-positive cells. At this stage, the organoid consists of multiple rosette-like arrangements of SOX2-positive neuroepithelial cells surrounding fluid-filled spaces. Similar to what we observed in the 2D NPC cultures, PANX1 staining shifted at this stage to concentrate at the apical membrane region of these neural rosettes (Fig. 3C, arrowheads). As apical-basal polarity becomes fully established at the end of neuroepithelial expansion (day 10), PANX1 is preferentially localized toward the apical surface of each neuroepithelial sphere as shown through whole-mount immunofluorescence (Fig. 3D). To complement the whole-mount immunofluorescence data presented in Fig. 3B–D, we cryosectioned day 10 organoids and performed immunofluorescence confocal imaging of PANX1 at the neuroepithelial expansion stage of cortical organoid development. Confocal imaging of cryosectioned day 10 organoids confirmed a striking concentration of PANX1 protein localized to the apical edge of the neuroepithelial buds (Fig. 3E). Together, these data indicate that PANX1 is expressed throughout the embryonic stages of cerebral organoid development, from the beginning of neural lineage commitment to the formation of polarized neuroepithelium where the channels largely reside at the apical edge.

### PANX1 genetic ablation and pharmacological inhibition results in significantly smaller organoids

**Fig. 4:**
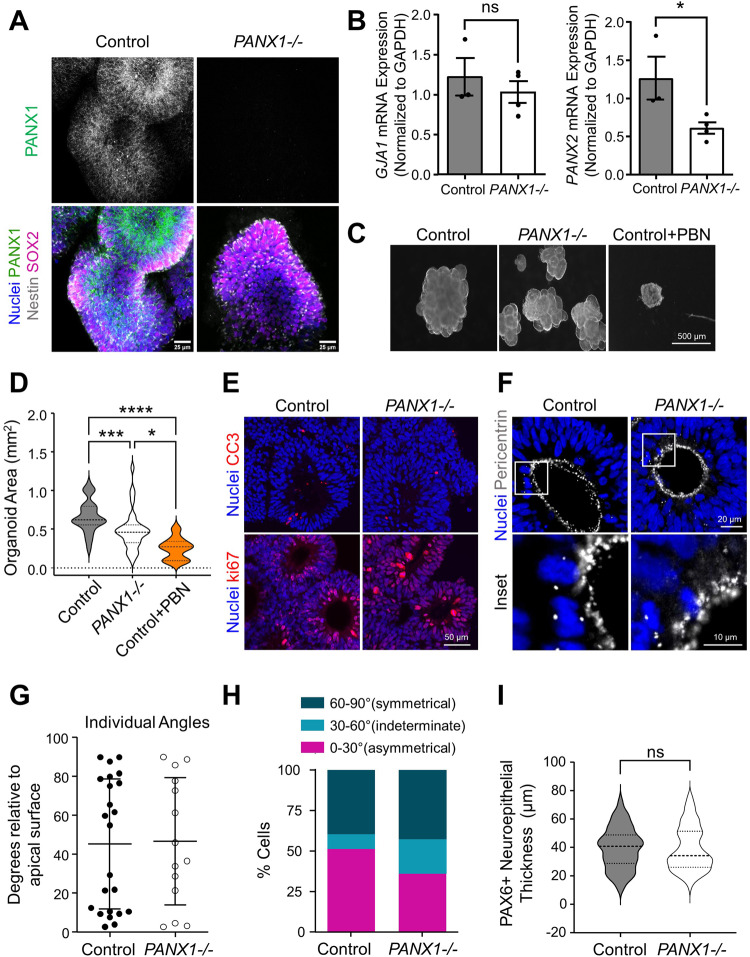
PANX1 inhibition results in significantly smaller organoids at neuroepithelial expansion stage. **A** Whole-mount immunofluorescence confocal imaging of PANX1 (green) in control and *PANX1−/−* day 10 cerebral organoids. Nestin (gray), SOX2 (magenta), nuclei (Hoechst, blue). **B** qPCR evaluation of *GJA1* and *PANX2* gene expression in day 10 control and *PANX1−/−* organoids. Transcripts for *PANX3* were undetectable (data not shown). Each sample is normalized to *GAPDH* and represented as a fold-change relative to control organoids (ddCT). *N* = 3 for control and *N* = 4 for *PANX1−/−* organoids where *p < 0.05; ns nonsignificant. **C**, **D** Representative phase images and analysis of organoid area of control and *PANX1−/−* organoids at day 10 as well as control organoids treated with 1 mM probenecid (PBN) starting on day 5. *N* = 46 for untreated control organoids, *N* = 34 for untreated *PANX1−/−* organoids, *N* = 9 for control probenecid-treated organoids. *p < 0.05; **p < 0.01; ****p < 0.0001. **E** Representative immunofluorescence confocal micrographs of cleaved caspase 3 (CC3) or ki67 (red) and nuclei (Hoechst, blue) in day 10 control and *PANX1−/−* organoids. **F** Representative immunofluorescence confocal micrographs of pericentrin (gray) and nuclei (Hoechst, blue) in day 10 control and *PANX1−/−* organoids. Pericentrin reveals the angle of division in neuroepithelial cells at the apical domain of control and knockout organoids. **G**, **H** Categorization of cell division angles relative to the apical surface. Angles of 0–30° indicate horizontal cleavage (asymmetric divisions and differentiation of the daughter cell). Angles of 30–60 degrees are indeterminate. Angles of 60–90° indicate vertical cleavages (symmetrical divisions resulting in NPC renewal). **I** Thickness of PAX6+ neuroepithelial cells in control and *PANX1−/−* day 10 organoids (from apical to basolateral edge). Control = 81 FOV; *PANX1−/−* = 84 FOV. Scale bars as indicated.

At neuroepithelial expansion, NPCs proliferate rapidly via symmetric division to make up the required tissue and organ mass. After expansion, NPCs must successfully migrate and differentiate via asymmetric division into mature neural cell types. We have previously reported that *PANX1−/−* iPSCs exhibit deficits in ectoderm lineage specification [12]. Given the upregulation of PANX1 during NPC differentiation, we hypothesized that loss of PANX1 would compromise the neuroepithelial expansion stage of organoid development. Immunofluorescence imaging of whole mount day 10 organoids confirmed the absence of PANX1 in our CRISPR-Cas9 knockout organoids (Fig. 4A). We used qPCR to assess whether *PANX1* ablation elicits compensatory upregulation of other pannexin isoforms (*PANX2* and *PANX3*) or the gap junction channel connexin43 (*GJA1*) in day 10 organoids (Fig. 4B). Transcripts for *PANX3* were undetectable and *GJA1* was not statistically different between control and *PANX1−/−*. Interestingly, *PANX2* mRNA transcripts were downregulated in the *PANX1−/−* organoids. *PANX1−/−* organoids were significantly smaller than control, which was even more pronounced in probenecid (PBN)-treated organoids (Fig. 4C, D). Possible reasons for smaller organoids at neuroepithelial expansion include differences in apoptosis, proliferation, or an imbalance in symmetric/asymmetric cell division. To that end, we found similar proportions of cleaved caspase 3 (apoptosis marker) and ki67 (proliferation marker) in our control and *PANX1−/−* organoids, suggesting little difference in apoptosis or cell proliferation (Fig. 4E). Cell division angle relative to the apical surface can reveal whether NPCs will undergo symmetrical division (self-renewal) or asymmetrical division (differentiation) [18]. Because PANX1 is reported to positively regulate neural progenitor cell self-renewal and proliferation [6], we examined organoid size and thickness of the PAX6+ neuroepithelial progenitors in day 10 organoids. Skewed symmetrical/asymmetrical NPC division could result in premature neuronal differentiation, which could account for the small size of *PANX1−/−* organoids. However, we found no difference in the proportion of nuclei undergoing vertical (symmetrical) or horizontal (asymmetrical) divisions (Fig. 4F–H). Furthermore, the thickness of the PAX6+ progenitor cell layer was not significantly different in *PANX1−/−* organoids compared to control (Fig. 4I).

### Transcriptomic analysis of PANX1*−/−* organoids reveal gene expression changes related to neural development

**Fig. 5:**
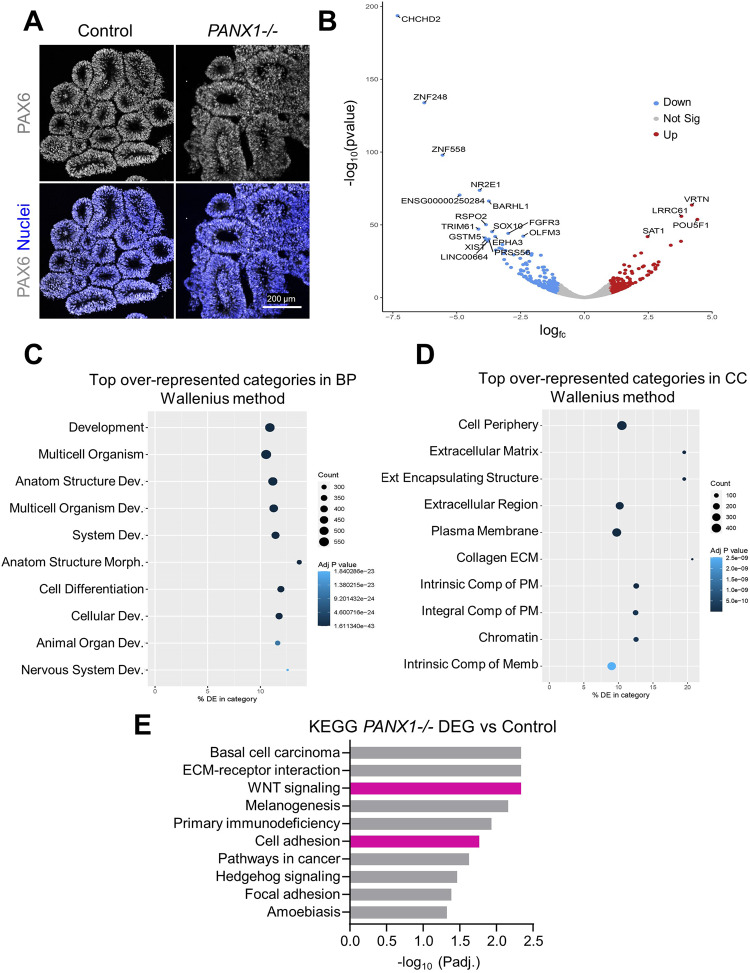
*PANX1−/−* organoids exhibit gene expression changes related to cell-cell adhesion, signaling, extracellular matrix and development. **A** Immunofluorescence confocal imaging of cryosectioned day 10 cerebral organoids demonstrating proportion of PAX6 positive (gray) neural progenitor cells. Scale bars as indicated. **B**–**E** Bulk RNA sequencing of control and *PANX1−/−* day 10 cerebral organoids. **B** Volcano plot illustrating the top differentially expressed genes where log_2_ fold change is >|1|. 231 genes were significantly UPregulated in *PANX1−/−* relative to control and 222 genes were significantly DOWNregulated. **C** GoSeq plot showing the top overrepresented categories in biological process. **D** GoSeq plot showing the top over-represented categories in cellular compartment. **E** KEGG pathway analysis for significant differentially expressed genes (DEG) in *PANX1−/−* organoids. Highlighted in pink are KEGG pathways associated with WNT signaling and cell adhesion.

Although PANX1 genetic ablation and pharmacological inhibition resulted in significantly smaller organoids at the neuroepithelial stage, none of the metrics we evaluated in Fig. 4 were changed in our *PANX1−/−* organoids. Therefore, we compared transcriptomic profiles of day 10 *PANX1−/−* organoids to control organoids using RNA-sequencing technology. Immunofluorescence imaging of cryosectioned day 10 organoids confirmed that nearly all of cells within day 10 cerebral organoids are PAX6 positive neural progenitor cells (Fig. 5A). Analysis of transcriptomic data using DESeq2 revealed a total of 1,047 differentially expressed genes with an adjusted *p*-value of less than 0.05. Limiting the genes to those with a log fold change (log_2_FC) of at least 1 (or −1) resulted in 453 differentially expressed genes (231 upregulated, 222 downregulated) in response to *PANX1* knockout (Fig. 5B; Supplementary Data). Similar to our previous study, pluripotency-related genes such as *POU5F1* (OCT4) and *ZSCAN10* were among the most significantly upregulated individual genes in *PANX1−/−* organoids compared to control [12]. Also observed was upregulation of vertebrae development-associated (*VRTN*), cell differentiation homeobox protein (*NKX1-2*) [19], and developmental factor Forkhead box H1 (*FOXH1*) [20]. The most significantly downregulated genes included the anti-apoptotic coiled-coil-helix-coiled-coil-helix domain containing 2 (*CHCHD2*) [21], the orphan nuclear receptor tailless (*TLX/NR2E1*) [22], signaling molecule R-spondin 2 gene (*RSPO2*) [23] and neuronal homeobox genes *BARHL1* and *BARHL2* [24].

Gene set enrichment analysis (GSEA) using gene ontology (GO) terms related to biological processes (BP), molecular function (MF), cellular component (CC), and KEGG pathways was used to group differentially expressed genes along common biological themes (Fig. 5C-E; Supplementary Data). Most GO:BP categories included genes involved in developmental processes (Fig. 5C) while GO:MF categories were over-represented by genes associated with cell signaling. In terms of cellular components, differentially expressed genes were over-represented in GO categories related to plasma membrane components as well as extracellular and peripheral cellular regions (Fig. 5D). GSEA analysis allowed us to categorize differentially expressed genes according to KEGG pathway maps representing what is currently known about molecular interactions and biological networks. We identified 10 KEGG pathways that were significantly over-represented in our GSEA analysis (adjusted p-value < 0.05) (Fig. 5E and Supplementary Data). Among the most over-represented KEGG pathways are those associated with ECM-receptor interactions and adhesion molecules (KEGG categories 04512, 04514 and 04510) (Table 1). Signaling pathways related to WNT signaling and Hedgehog signaling were also represented in our differentially expressed genes (KEGG categories 04310 and 04340) (Table 2). Finally, we found a surprising number of non-coding RNA molecules differentially expressed in our *PANX1−/−* organoids compared to control (Table 3).

**Table 1:** Differentially expressed adhesion genes in day 10 *PANX1−/−* organoids.

Adhesion Up	Fold increase	Role in neurodevelopment	Refs
*PCDHGB4*	3.011	• Pathogenic sequence variations related to neurodevelopmental disorder with poor growth and skeletal anomalies • Involved self-recognition and avoidance	[57]
*CD-40*	2.603	• Hippocampal excitatory neuronal dendrites stunted in Cd40−/− mice • CD40 isoforms temporally modulate neuron differentiation during brain development	[58, 59]
*CNTN2*	2.392	• Axonal elongation, axonal guidance, and cellular migration • CNTN2/TAG-1 deficient mice exhibit a significantly smaller cortex and a reduction of corticothalamic axons	[60, 61]
*PCDHGA10*	2.356	• Pcdhg isoforms regulate cortical inhibitory interneuron survival during the endogenous period of programmed cell death	[62, 63]
*CLDN6*	2.229		
*PCDH8*	2.204	• Pcdh8 knockout mouse neurons exhibit increased dendritic spines in the presence of N-cadherin	[64, 65]
*CD4*	2.015		
*PCDHA10*	2.007	• Pcdhg isoforms regulate cortical inhibitory interneuron survival during the endogenous period of programmed cell death	[62, 63]

Adhesion Down	Fold decrease	Role in neurodevelopment	Refs
*PCDH7*	2.557	• Promotes neural differentiation and dendritic spine morphology in zebrafish and mouse development	[66, 67]
*CDH15*	2.353	• Linked to ASD and Intellectual Disability	[68, 69]
*VCAM1*	2.128	• Present in adult hippocampal neural stem cell subpopulation • Vcam1−/− mice exhibit impaired spatial learning and memory in mice and fewer proliferating NSCs	[70]
*CDH9*	2.108	• GWAS association with ASD	[71]
*CDH19*	2.047		
*NCAM2*	2.015	• Implicated in neurodevelopmental disorders including Down syndrome and autism • Promotes filopodia formation and neurite branching • NCAM2 downregulation leads to dendritic branching defects	[72, 73]
*CLDN1*	1.805	• Patients with CLDN1 mutations may be an increased risk for learning disabilities, mental retardation, and language delay	[74]
*PCDHA11*	1.733	• Pcdhg isoforms regulate cortical inhibitory interneuron survival during the endogenous period of programmed cell death	[62, 63]
*MPZ*	1.331	• Adhesion molecule necessary for normal myelination in the peripheral nervous system • Associated with Charcot‐Marie‐Tooth neuropathy type 1 B (CMT1B)	[75, 76]

**Table 2:** Differentially expressed WNT pathway genes in day 10 *PANX1−/−* organoids.

WNT up	Fold increase	Role in neurodevelopment	Refs
*WNT8A*	5.49	• Wnt8 inhibits BMP4 expression, allowing neural induction from ectoderm • Promotes synaptogenesis via LRP6	[77, 78]
*DKK4*	3.61	• Associated with schizophrenia	[79]
*FZD10*	2.52	• Plays a role in neural tube patterning	[80]
*PRKG2*	2.49	• PRKG2-deficient mice exhibit restricted growth and deficits in learning and memory, similar to human patients	[81]
*RSPO4*	2.23		

WNT down	Fold decrease	Role in neurodevelopment	Refs
*RSPO2*	14.47	• Promotes midbrain dopaminergic neurogenesis and differentiation	[23]
*WNT2B*	4.91	• Associated with bipolar disorder	[82]
*WNT7A*	4.72	• Stimulates neural stem cell proliferation and promotes neuronal differentiation • Associated with bipolar disorder	[82–84]
*RSPO3*	4.22		
*RSPO1*	2.99		
*SOST*	2.34		
*WNT7B*	2.26	• Promotes dendrite development in mouse hippocampal neurons	[85, 86]
*WNT8B*	2.15	• Expression restricted to the developing forebrain	[87]
*WNT3A*	2.01

**Table 3:** Differentially expressed non-coding RNAs in day 10 *PANX1−/−* organoids.

ncRNA Up	Fold increase	Role in neurodevelopment	Refs
*JAKMIP2-AS1*	5.74	• High expression in neural precursor cells	[88]
*MIR302CHG*	3.46	• MiR-302/367 cluster host regulates neural progenitor proliferation, differentiation and survival during neurulation	[89]
*PCAT14*	3.16		
*LINC01356*	3.00		
*LINC00958*	2.41		
*LINC00649*	2.39		
*MIR100HG*	2.27		
*LINC02381*	2.15		
*LNCTAM34A*	2.11		
*SATB1-AS1*	2.10	• Antisense for SATB1 (TF associated with neuronal maturation and cortical development) • Associated with Anxiety disorder	[90, 91]
*XACT*	2.06	• Regulates neuronal differentiation in both male and female hPSCs	[92]
*LINC01415*	2.01		
*LINC01833*	2.01		

**ncRNA Down**	**Fold decrease**	**Role in neurodevelopment**	**Refs**
*XIST*	14.49	• XIST dosage correction of Trisomy 21 promotes NSC differentiation to neurons	[93]
*LINC00664*	13.77		
*LINC01515*	5.62	• Dysregulated in autism spectrum disorder patients	[94]
*SVIL-AS1*	5.50		
*MIR9-1HG*	4.63	• Potential regulator of primate ganglionic eminences	[95]
*FAM218A*	4.54		
*FLG-AS1*	4.21		
*MIR9-3HG*	2.63		
*LINC00461*	2.53	• Pleiotropic effects on five psychiatric traits; important for mouse neurodevelopment	[96]
*OSTM1-AS1*	2.50		
*MIR1-1HG-AS1*	2.44		
*MIR924HG*	2.43		
*NR2F1-AS1*	2.30	• Pro-neurogenic; genetic disruption contributes to complex neurodevelopmental disorders.	[97]
*LHX5-AS1*	2.06	• Strongly enriched in early human embryonic brain, becoming restricted to the developing cortical plate in stage 14–16 embryos	[98]

### PANX1 co-localizes with apically situated junctional proteins at the neuroepithelial expansion stage of cerebral organoids

**Fig. 6:**
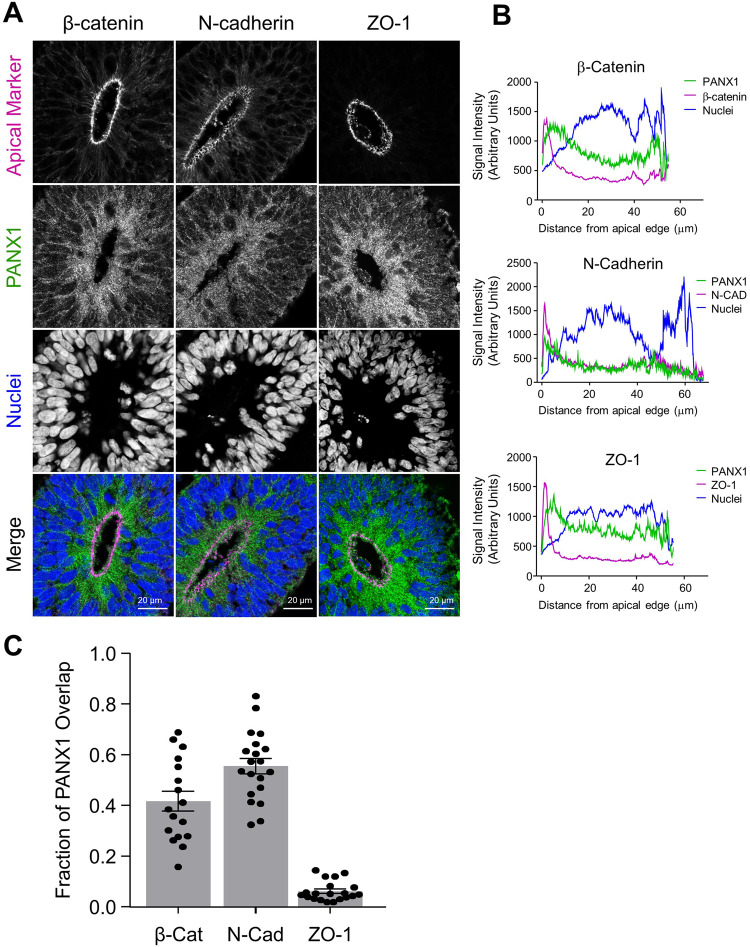
PANX1 co-localizes with key junctional proteins at the apical edge of neuroepithelial cells in cerebral organoids. **A** Representative immunofluorescence confocal images demonstrating PANX1 (green) colocalization with the apical membrane proteins β-catenin, N-cadherin, and ZO-1 (magenta) at the apical side (innermost) of the neuroepithelia. Scale bars as indicated. **B** Line graphs and **C** Manders’ correlation coefficients depicting the fraction of signal overlap for PANX1 with apically situated adherens junctions β-catenin and N-cadherin as well as with tight junction ZO-1. Error bars depict the standard error of the mean for 17–24 fields of view obtained from 4 independent experiments.

**Fig. 7:**
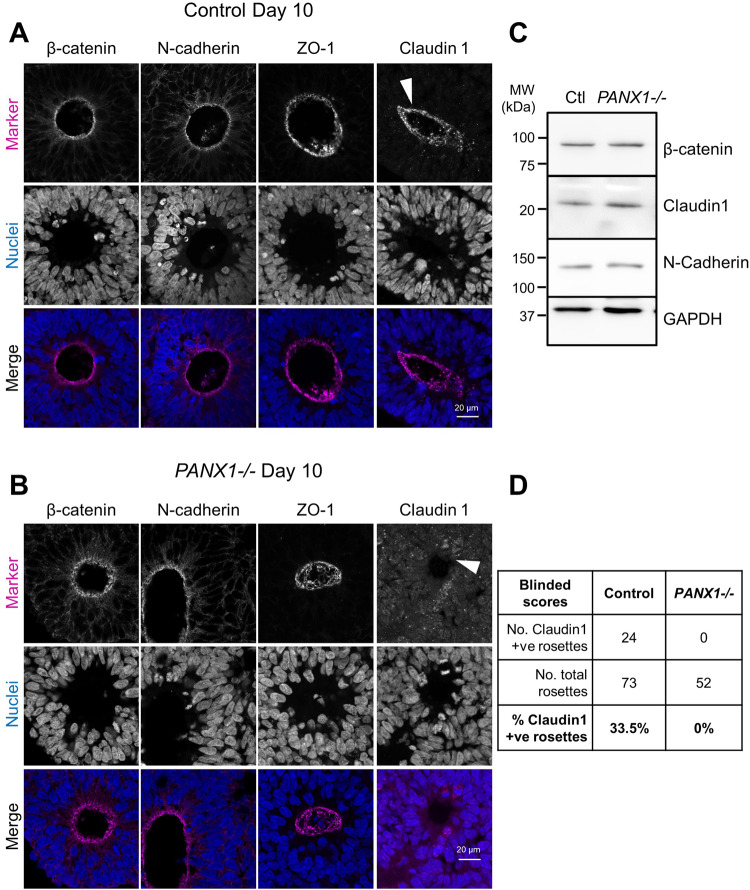
Apical adhesion molecule expression and localization unchanged in *PANX1−/−* cerebral organoids. **A, B** Representative immunofluorescence confocal micrographs for select apical markers (magenta) in day 10 control and *PANX1−/−* cerebral organoids. Nuclei (Hoechst, blue). Arrowhead indicates the redistribution of Claudin 1 away from the apical membrane domain in *PANX1−/−* organoids. Scale bars as indicated. **C** Western blots of apical protein expression in control and *PANX1−/−* day 10 cerebral organoids. **D** Blinded scoring of Claudin 1 localization to apical membrane domain in control and *PANX1−/−* day 10 cerebral organoids.

Given the abundance of altered genes related to cell adhesion and WNT signaling in our *PANX1−/−* organoids, and because β-catenin was recently recognized as a PANX1 interacting partner [25], we investigated key junctional protein targets at the apical domain to see if they are differentially expressed or localized in *PANX1−/−* organoids (Figs. 6 and 7). Rosette formation depends upon apical-basolateral patterning, coordinated by several key proteins including N-Cadherin, β-Catenin and ZO-1 [18]. Therefore, we next examined the colocalization of PANX1 with these apically situated adhesion proteins (Fig. 6). Manders’ correlation coefficients demonstrated a robust colocalization of PANX1 with β-catenin (0.4070 ± 0.06509) and PANX1 with N-cadherin (0.5457 ± 0.05012). However, there was very little colocalization between PANX1 and the tight junction protein ZO-1 (0.06847 ± 0.01272) (Fig. 6C). We next evaluated the expression and localization of β-catenin, Claudin 1, N-cadherin and ZO-1 in *PANX1−/−* organoids (Fig. 7). Although several of these gene classes were significantly altered in the RNAseq dataset, Western blotting and immunofluorescence showed similar expression and localization patterns between control and *PANX1−/−* organoids (Fig. 7A–C). Claudin 1 localization was a bit trickier as it was only observed at the apical membrane domain in ~33% of control neural rosettes (Fig. 7A, B, D). However, Claudin 1 was never present at the apical membrane domain in any *PANX1−/−* neural rosettes.

### PANX1 is preferentially expressed in neurons as cerebral organoids mature

**Fig. 8:**
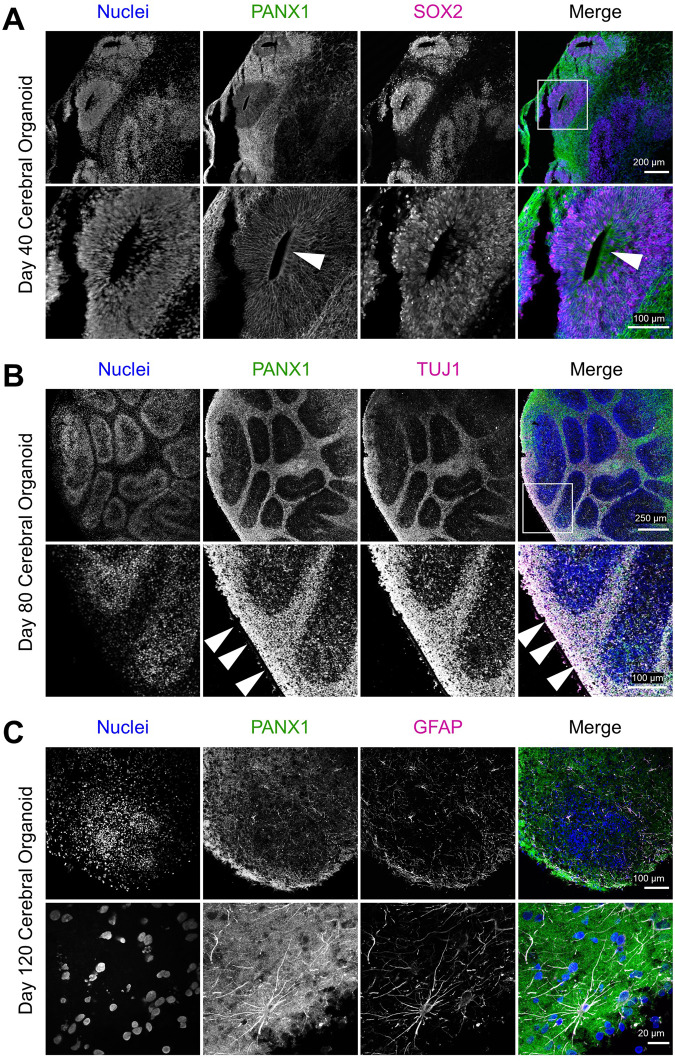
PANX1 expression in neurons and glia of mature cerebral organoids. Representative immunofluorescence confocal images showing PANX1 (green) localization to various regions and cell types throughout cerebral organoid maturation. **A** Forty-day-old cerebral organoids exhibit abundant PANX1 staining outside the SOX2-positive ventricular-like zones with limited PANX1 expression persisting at the ventricular-like zone’s apical edge (arrowhead, inset). **B** Day 80 cerebral organoids display bright PANX1 expression in areas where TUJ1-positive neurons reside (arrowheads, inset). **C** PANX1 expression is apparent in GFAP-positive glial cells with astrocyte-like morphology in 120-day-old cerebral organoids. Nuclei (Hoechst, blue). Neural cell type markers (SOX2, TUJ1, and GFAP in magenta). Scale bars as indicated.

As cerebral organoids mature, the numerous neural rosettes continue to hollow out and elongate, forming fluid-filled ventricular-like spaces. Furthermore, the tightly packed neuroepithelia and NPCs residing in the emerging ventricular-like zone begin to asymmetrically divide and migrate, ultimately differentiating into neurons, and later, to glia. To examine how PANX1 expression and localization change as organoids begin to mature and establish cortical layering, we examined mature cerebral organoids between 40 to 120 days (Fig. 8). Immunostaining revealed some apical expression of PANX1 along SOX2-positive ventricular-like zones in day 40 organoids (Fig. 8A). However, PANX1 signal intensity appeared brightest outside the ventricular-like zones coinciding in regions with TUJ1-positive neurons (Fig. 8B). In 120-day old organoids we observed PANX1 expression within stellate GFAP-positive cells with astrocyte-like morphology (Fig. 8C). Our results indicate that mature organoids exhibit PANX1 localization at the apical side of the ventricular-like zones and in more developmentally advanced neural cell types such as neurons and GFAP-positive glia.

## Discussion

Given that PANX1 is expressed in the earliest cell types of human development and is linked to neurological disease, we sought to explore PANX1 expression and localization throughout early stages of human brain development. Although most PANX1 studies focus on perinatal or adult mouse models, PANX1 is expressed in human oocytes, embryos and pluripotent stem cells, suggesting a fundamental role for PANX1 in human development [10–12]. The potential role of PANX1 in human brain development is further supported by a loss-of-function human germline *PANX1* variant in a patient with severe neurological deficits [2], and by *PANX1* and *PANX2* single nucleotide polymorphisms implicated in autism spectrum disorder [1]. Indeed, we recently reported that PANX1 is expressed in human induced pluripotent stem cells (iPSCs) and our *PANX1−/−* iPSCs exhibit decreased ectoderm formation compared to control [12]. This finding indicated to us that PANX1 might impact the development of ectodermal-derived tissues, such as the brain. The Allen Institute’s Brainspan prenatal laser microdissection microarray dataset depicts PANX1 transcript expression in 21 pcw human fetal brains (Brainspan.org). Because transcript expression does not always correlate to protein expression, we were surprised that we could not find published evidence of PANX1 protein expression at this stage of human development. We have now confirmed that PANX1 protein is also expressed across all layers of the developing human cerebral cortex with brighter manifestation in the marginal zone and subventricular zone. The high PANX1 expression in the human fetal brain, combined with our findings of ectodermal lineage deficits in human *PANX1−/−* iPSCs suggests a role for PANX1 in human neural development.

We previously reported that PANX1 is expressed at the cell surface across iPSCs and iPSC-derived embryoid bodies [12]. Intriguingly, we find here that PANX1 localization becomes very restricted upon cerebral organoid neural induction, when organoids begin to arrange into neural rosettes (Figs. 2, 3, 6). As apical-basolateral polarity becomes fully established at the end of neuroepithelial expansion, the organoid consists of multiple rosette-like arrangements of SOX2-positive neuroepithelial cells surrounding fluid-filled spaces. These neuroepithelia are the progenitor cells of the developing brain and express tight junctions, adherens junctions, and exhibit apical-basolateral polarity, forming a layer of pseudostratified columnar neuroepithelium which gives rise to the neural plate and subsequent neural tube [18, 26, 27]. At the neuroepithelial expansion stage of cerebral organoid development, PANX1 was preferentially localized toward the apical surface of each neuroepithelial rosette and colocalized with key apical proteins including β-catenin and N-cadherin (Fig. 6). Our RNAseq in day 10 organoids revealed a significant downregulation in several adhesion molecules including cadherins, claudins, neural cell adhesion molecule 1 (NCAM), and others. In addition to establishing polarity in the developing brain, these cytoskeleton-anchoring proteins help to coordinate the mitotic spindle orientation and several downstream signal transduction cascades controlling neural cell fate. In the future it would be interesting to investigate whether PANX1 physically interacts with these junctional proteins as has been recently shown in other systems [25]. In addition to being significantly upregulated in neurons compared to iPSCs, our Western blot analyses showed differential PANX1 banding patterns between iPSCs, NPCs, and neurons where most of the PANX1 in NPCs and neurons exists as a high molecular weight species. These different molecular weight species have been shown to correspond to different PANX1 glycosylation species with unglycosylated (Gly0), simple glycosylation (Gly1) and complex carbohydrate modifications (Gly2), which are thought to influence plasma membrane targeting [28]. Other groups have shown that PANX1 may be preferentially distributed to basolateral or apical membrane compartments depending on the cell and tissue type where intracellular PANX1 retention inhibits cell polarization [29, 30]. Apical PANX1 channels residing at the unopposed edge of the ventricular-like zone may have implications for paracrine signaling and long-range coordination of NPC proliferation in the ventricular-like zone. In the postnatal murine brain, ATP released into the extracellular space by PANX1 channels activates P2X7 and P2Y purinergic receptors which in turn stimulate the proliferation of NPCs [6]. Future studies will determine whether PANX1 serves a similar role in neuroepithelial expansion at this early stage of development.

Current literature implicates PANX1 with WNT/β-catenin signaling through the physical interaction of PANX1 with β-catenin in melanoma cells [25, 31]. PANX1 pharmacological inhibition or gene knockdown slowed melanoma cell growth and significantly decreased β-catenin protein levels in melanoma cells. Here we find that PANX1 and β-catenin are both apically localized in day 10 neuroepithelial rosettes and exhibit considerable colocalization. However, we detected no change in cell proliferation or apoptosis in our *PANX1−/−* organoids, nor did we find any appreciable changes in β-catenin localization or expression with *PANX1* genetic ablation. In the brain, WNT signaling inhibits the self-renewal of murine cortical neural precursor cells and promotes differentiation of neuronal cell types such as dopaminergic neurons [23, 32]. Indeed, several of the differentially expressed WNT-associated genes in our *PANX1−/−* organoids are associated with neurodevelopment and neural stem cells. Thus, PANX1 plays an important role in WNT signaling for both melanoma cells and neuroepithelial cells, albeit through different mechanisms.

Our RNAseq screen revealed a surprising number of differentially expressed non-coding RNAs (ncRNAs). Very few studies to date have linked PANX1 with ncRNA expression [33, 34]. Not all of the ncRNAs that came out in our screen have documented roles in neurodevelopment, however several are associated with neural stem cells, neuronal differentiation and neurodevelopmental disorders. Many of the ncRNAs in our screen are related to cell cycle control and apoptosis in different forms of cancer. Given the smaller size of our *PANX1−/−* day 10 organoids, it is possible that differential expression of ncRNAs could contribute to changes in neural precursor cell proliferation or apoptosis. However, as we saw no obvious changes in ki67 (cell proliferation) or cleaved caspase 3 (apoptosis) expression in our *PANX1−/−* organoids, we suspect the smaller organoids are not related to changes in cell proliferation or apoptosis.

Despite being apically localized at the neuroepithelial expansion stage, PANX1 resided primarily in TUJ1-expressing neurons in mature cerebral organoids, with lesser amounts persisting in the SOX2-positive ventricular-like regions (Fig. 8). Consistent with this observation, PANX1 protein expression was significantly elevated in differentiated neurons compared to NPCs (Fig. 2). We found a similar pattern in the human fetal cortex where PANX1 staining was concentrated outside of the ventricular zone (Fig. 1). This is in contrast to reports in mouse brains where PANX1 was found to be concentrated in periventricular neural stem cells in postnatal day 15–60 mice [6, 7, 35]. Just as we found a dramatic shift in PANX1 cellular distribution between day 10 and 40 cerebral organoids, it is possible that PANX1 could again change distribution between the fetal and adult brains.

In postnatal murine brains, pharmacological inhibition of PANX1 channels prevents NPC proliferation and enhances neuronal differentiation by promoting neurite extension and cell migration [6, 7]. Others have demonstrated PANX1 localization at neuronal synapses where the channels help to replenish extracellular ATP, negatively regulate dendritic spine density, and maintain synaptic strength [8, 9, 36]. It remains to be seen whether human iPSC-derived *PANX1−/−* neurons exhibit similar increases in spine and branching density as has been observed in mouse. Another major Pannexin isoform, Panx2, has shown similar roles in NPC maintenance as *Panx2* knockdown in Neuro2a cells significantly accelerated neuronal differentiation [37]. We report here that our stunted *PANX1* knockout cerebral organoids exhibit a significant decrease in *PANX2* mRNA. Moreover, treating the organoids with probenecid, which should pharmacologically inhibit both PANX1 and PANX2 channels caused an even more pronounced decrease in organoid size. Thus, the stunted neuroepithelial expansion we observed in our *PANX1−/−* organoids might be due to a loss of both PANX1 and PANX2 proteins.

Cerebral organoids are emerging as a valuable tool to model early neurodevelopmental processes and developmental disorders such as autism spectrum disorder, microcephaly and others [38]. Human iPSC-derived organoids are also amenable to CRISPR-Cas9 genetic manipulation to induce gene knockout or insert pathogenic variants or variants of unknown significance. This makes organoids a valuable tool to uncover how individual genes (such as *PANX1*) influence human development and disease in a human background. Because they can be derived from individual patients, they are also a useful platform for evaluating drug toxicity and therapeutic drug screening toward precision medicine. Cerebral organoids have several advantages over traditional mouse models, including the ability to observe much earlier developmental timepoints, the relative ease of genetic and pharmacological manipulation, and being comprised of human cells. However, a caveat of human cerebral organoids is the absence of microglia and blood vessels, which are thought to emerge during gestational weeks 4–24 [39]. Here, we primarily focused on neuroepithelial expansion, which mimics the neurulation stage of development (gestational week 3–4), just before microglia and blood vessels would have developed in utero. We conclude that PANX1 is dynamically expressed by multiple cell types in the developing human cerebral cortex. In combination with previous reports from our group and others, this study details the participation of PANX1 in iPSC lineage restriction, co-localizations with key apical membrane proteins and junctional complexes in neuroepithelial rosettes, and PANX1 upregulation and redistribution to TUJ1-expressing neurons within mature human cerebral organoids.

## Materials & methods

### Induced pluripotent stem cells

These studies were approved by the Newfoundland and Labrador Health Research Ethics board (HREB # 2018.210). A male iPSC line (GM25256) was purchased from the Coriell Institute for Medical Research (Cat# GM25256, Coriell, Camden, NJ, USA). The female iPSCs were created as described previously [40] and obtained through a material transfer agreement with The University of Western Ontario. Both cell lines were derived from fibroblasts of apparently healthy individuals with no known genetic pathologies.

iPSCs were cultured in a humidified 37 °C cell culture incubator buffered with atmospheric oxygen and 5% CO_2_. The iPSCs were grown on Geltrex™-coated (Cat# A141330, ThermoFisher, Waltham, MA, USA) culture dishes and fed daily with Essential 8™ medium (Cat# A1517001, ThermoFisher) or mTeSR™ Plus (Cat #100-0276, STEMCELL Technologies, Vancouver, BC, CAN) maintenance medium. Every 4–5 days, iPSCs were passaged as small aggregates using a cell scraper and 0.5 mM EDTA (Cat #AM9260G, ThermoFisher) prepared in Ca^2+^/Mg^2+^-free phosphate buffered saline (PBS; Cat# 319-005-CL, WISENT Inc., St. Bruno, QC, CAN) [41] when the colonies exhibited smooth borders and tight cell packing. Aggregates were seeded into fresh Geltrex™-coated wells containing Essential 8™ or mTeSR™ Plus at split ratios of 1:5 to 1:50. StemPro™ Accutase™ (Cat# A1110501, ThermoFisher) was used to create suspensions of single cell iPSCs. Single cells were plated in medium supplemented with 10 µM of the rho-associated kinase inhibitor (ROCKi), Y-27632 (Cat# 100005583, Cayman Chemicals, Ann Arbor, MI, USA) to promote single cell iPSC survival [42]. After thawing from liquid nitrogen stocks, iPSCs were maintained in culture for up to 20 weeks at which point a new vial was thawed. Evaluation of our iPSC cell banks with the hPSC Genetic Analysis Kit (Cat # 07550, STEMCELL Technologies) confirmed normal copy number at various mutation hotspots and assessment with a Mycoplasma PCR Detection Kit (Cat# G238, Applied Biological Materials Inc., Richmond, BC, CAN) indicated that cell stocks are free of mycoplasma.

*PANX1−/−* iPSCs were created using CRISPR-Cas9 as previously described [12]. Briefly, iPSCs were transfected with the pSpCas9(BB)−2A-GFP plasmid (Cat# 48138, Addgene, Cambridge, MA, USA) [43] containing the *PANX1*-specific sgRNA: 5′-GCTGCGAAACGCCAGAACAG-3′. GFP-expressing single cells were sorted using fluorescence activated cell sorting (FACS) and individual clones were examined for *PANX1* ablation via Sanger sequencing and Western blotting.

### Monolayer Differentiation to Neural Progenitors and Neurons

Human iPSCs were differentiated to neural progenitor cells according to the methodology described by [44] with several modifications. On day 0, singularized iPSCs were plated at a density of 200,000 viable cells/cm^2^ onto Geltrex™-coated dishes containing Gibco™ PSC Neural Induction Medium (Cat# A1647801, ThermoFisher) supplemented with 10 µM ROCKi. Daily feeds with PSC Neural Induction Medium without ROCKi were administered until day 7 when the cells were singularized and re-plated at a density of 200,000 viable cells/cm^2^ onto Geltrex™-coated dishes containing Neural Stem Cell (NSC) Expansion Medium supplemented with 10 µM ROCKi. NSC Expansion Medium consists of 49% Neurobasal (Cat# 21103049, ThermoFisher), 49% Advanced DMEM/F12 media (Cat# 12634010, ThermoFisher), and 2% (1X) Neural Induction Supplement (Cat# A16477-01, ThermoFisher). Cells were fed daily with NSC Expansion Medium without ROCKi and seeded into new Geltrex™-coated wells every 7 days. On day 21 or 22 the resultant NPCs were assayed or differentiated further to neurons.

For differentiation to neurons, day 21 or 22 NPCs were passaged as single cells and seeded at 50,000 cells/cm^2^ onto culture wells coated with 10 µg/mL laminin (Cat# 354232, Corning Inc, Corning, NY, USA) containing Neuron Differentiation Medium supplemented with 10 µM ROCKi. Neuronal Differentiation Medium consists of ~96% Neurobasal (Cat # 21103049, ThermoFisher), 2% (1X) B-27 (Cat #17504044, ThermoFisher), 1% (1X) non-essential amino acids (Cat# 321-011-EL, WISENT), 20 ng/mL brain-derived neurotropic factor (BDNF; Cat# 78005, STEMCELL Technologies), 20 ng/mL glial cell-derived neurotropic factor (GDNF; Cat# 78058, STEMCELL Technologies), and 200 µM l-ascorbic acid 2-phosphate sesquimagenesium salt hydrate (Cat# A8960, MilliporeSigma, Burlington, MA, USA). Half medium changes with Neuron Differentiation Medium were performed every other day for 14 days.

### Cerebral organoids

Cerebral organoids were generated using the STEMdiff™ Cerebral Organoid Kit and STEMdiff™ Cerebral Organoid Maturation Kit (Cat# 08570 & 08571, STEMCELL Technologies) according to the manufacturer’s instructions with the following modifications: On Day 0, 96-well round-bottom plates (Cat# 351177, Corning) were rinsed with a solution of 5% Pluronic™ F-127 (Cat# P2443, MilliporeSigma) prepared in deionized water to confer an anti-adherent coating [45]. On Day 7 the organoids were subjected to high throughput Geltrex™ embedding in Expansion Medium according to Chew et al., with slight modification [46]. Briefly, ice-cold liquid Geltrex™ was added at 1:50 dilution to ice-cold Expansion Medium. The organoids were quickly transferred into the cold Expansion Medium with Geltrex™ and re-plated into a fresh Pluronic™ F-127-coated 6-well dish (Cat# 140685, ThermoFisher).

### Human fetal brain preparation

These studies were approved by the Newfoundland and Labrador Health Research Ethics board (HREB # 2014.216). Formalin fixed and paraffin embedded samples from a 21–22 pcw human fetal brain were cut to a thickness of 5 µm using a microtome and deposited onto positively charged glass slides (Cat# ER4951PLUS, FisherScientific). The sections were dewaxed with xylene substitute (MilliporeSigma, Cat# 78475) and rehydrated with graded ethanol solutions. After rehydration, the sections were subjected to antigen retrieval and antibody staining for immunofluorescence.

### Immunofluorescence imaging

Monolayer cultures grown on Geltrex™ or laminin-coated #1.5 glass coverslips were fixed in 10% buffered formalin (Cat# CA71007-344, VWR, Radnor, PA, USA) for 10 min at room temperature and permeabilized with PBS-T (Ca^2+^/Mg^2+^-free PBS + 0.1% TWEEN® 20 (Cat# BP337-500, FisherScientific, Waltham, MA, USA)) for 20 min followed by 0.1% Triton™ X-100 (Cat# T5832, MilliporeSigma) in Ca^2+^/Mg^2+^-free PBS for 10 min. Samples were incubated overnight at 4 °C in primary antibodies diluted in PBS-T with 3% bovine serum albumin (BSA; Cat# 800-095-EL, WISENT Inc.) and 0.1% NaN_3_ according to Table 4. Secondary antibodies and/or dyes (Table 4) prepared in PBS-T were applied for 2 h at room temperature. All Alexa Fluor® and HRP (horseradish peroxidase) conjugated secondary antibodies were purchased from ThermoFisher. Slides were mounted using Mowial®488 reagent with 1,4-diazabicyclo[2.2.2]octane (DABCO) antifade compound according to the formulation described by Cold Spring Harbor [47]. For whole-mount imaging, fixed organoids were permeabilized and stained according to the methodology described above and transferred to an 8-well µ-slide high-end microscopy chamber slide (Cat# 80826, ibidi, Gräfelfing, DEU) for confocal imaging.

**Table 4:** Antibodies and dyes for immunofluorescence (IF) and western blot.

Marker	Supplier	Catalog #	Host species	IF	Western blot
β-Catenin	Santa Cruz (Dallas, TX, USA)	sc-7963	Mouse	1:500	1:2000
β-III Tubulin (TUJ1)	R&D Systems (Minneapolis, MN, USA)	MAB1195	Mouse	1:200	
Claudin 1	Cell Signaling Technologies (Danvers, MA, USA)	13995	Rabbit	1:200	1:1000
Cleaved Caspase 3 (Active)	BD Biosciences (Franklin Lakes, NJ, USA)	559565	Rabbit	1:500	
GAPDH	MilliporeSigma	MAB374	Mouse		1:5000
GFAP	Cell Signaling Technology	3670	Mouse	1:300	
Ki67	Abcam (Cambridge, UK)	ab16667	Rabbit	1:250	
MAP2	MilliporeSigma	M9942	Mouse	1:500	
N-Cadherin	BD Biosciences	610921	Mouse	1:500	1:2000
Nestin	ThermoFisher	14-9843-82	Mouse	1:500	
OCT4 BrilliantViolet™421	BioLegend (San Diego, CA, USA)	653712	Mouse	1:40	
PANX1	Laird Lab [99]	N/A	Rabbit	1:500	1:2000
PAX6 PerCP-Cy™5.5	BD Biosciences	562388	Mouse	1:100	
Pericentrin	Abcam	ab4448	Rabbit	1:1000	
SOX2	R&D Systems	AF2018	Goat	1:200	
ZO-1	ThermoFisher	33-910	Mouse	1:500	
Hoechst 33342	FisherScientific	H3570	Dye	1:1000	
Phalloidin-555	ThermoFisher	A34055	Dye	1:500	
Phalloidin-647	Cell Signaling Technology	8940	Dye	1:20	
To-Pro™−3 Iodide (642/661)	ThermoFisher	T3605	Dye	1:1000	

Cerebral organoids were fixed overnight (~20 h) in 10% normal buffered formalin and cryogenically prepared according to the methodology described in STEMCELL Technologies’ Document #27171, Version 1.0.0, Nov 2019. Briefly, organoids were first dehydrated in Ca^2+^/Mg^2+^-free PBS supplemented with 30% sucrose for 1–4 days at 4 °C until the organoids sank. Dehydrated organoids were then incubated for 1 h at 37 °C in gelatin embedding solution consisting of 10% sucrose and 7.5% gelatin (Cat# G1890, MilliporeSigma) prepared in Ca^2+^/Mg^2+^-free PBS. The organoids were then snap frozen in a slurry of dry ice and isopentane followed by cryosectioning at thickness of 14 µm and deposition onto positively charged glass microscope slides (Cat# ER4951PLUS, FisherScientific). For antigen retrieval, sections were placed into a plastic container with pH 6.0 citrate buffer: 0.294% Tri-sodium citrate (dihydrate) (Cat# A12274, Alfa Aesar, Tewksbury, MA, USA) + 0.05% TWEEN® 20 and heated in a food steamer (Hamilton Beach, Glen Allen, VA, USA) for 20 min. Immunostaining and mounting were performed as stated above with antibodies and dyes listed in Table 4.

### Phase contrast imaging

Phase contrast images of monolayer cells and organoids were taken on a Zeiss AxioObserver microscope using 5X/0.12 NA A-Plan and 10X/0.25 NA Ph1 objectives. Images from these microscopes were taken in 8-bit greyscale using an Axiocam MRm camera and AxioVision Version 4.8.2 software. All phase contrast imaging equipment is from Carl Zeiss Microscopy (Jena, DEU).

### Organoid size measurements

Area measurements from phase contrast images of day 10 cerebral organoids were performed automatically using the batch macro code in FIJI open source software [48]. Area measurements from images that contained debris (fibers and unincorporated cells) were performed manually by tracing around the object’s periphery and excluding debris protuberances. The macro shown here computes object area for entire folders of phase contrast images that were taken on the same microscope, at the same magnification. The macro can be adjusted for different magnifications and microscopes by changing the parameters in “Set Scale”.

macro “Batch Measure” {

 dir = getDirectory(“Choose a Directory “);

 list = getFileList(dir);

 if (getVersion > =“1.40e”)

 setOption(“display labels”, true);

 setBatchMode(true);

 for (i = 0; i < list.length; i++) {

 path = dir+list[i];

 showProgress(i, list.length);

 if (!endsWith(path,”/“)) open(path);

 if (nImages > =1) {

run(“8-bit”);

run(“Set Scale…”, “distance=388 known=500 pixel=1 unit=microns global”);

run(“Enhance Contrast…”, “saturated=0.3 normalize”);

run(“Auto Local Threshold”, “method=Phansalkar radius=15 parameter_1 = 0 parameter_2 = 0 white”);

run(“Analyze Particles…”, “size = 40000-Infinity display include add”);

selectWindow(“Results”);

 close();

 }

 }

}

### Confocal microscopy and image analysis

Fluorescent confocal images were primarily acquired on an Olympus Fluoview FV10i—W3 confocal microscope (Olympus, Tokyo, JPN) fitted with a 10X/0.4 NA or 60X/1.2 NA lens and Fluoview version 2.1.17 software. The following lasers were used to visualize fluorophores: Hoechst/Brilliant Violet™ 421 (405 nm laser); Alexa Fluor® 488 (473 nm laser); Alexa Fluor® 555/Phalloidin-555 (559 nm laser); Alexa Fluor® 647/Phalloidin-647/To-pro™−3 iodide (635 nm laser). Additional images were taken on an Olympus FV1000 confocal microscope fitted with 10×/0.4 NA, 20×/0.75 NA, 40×/0.95 NA or 60×/1.42 NA objectives and the following lasers: 405, 458, 568, and 633 nm. Tiled images of 21–22 pcw human cerebral cortex were taken on a ZEISS LSM 900 with Airyscan 2 fitted with 20X/0.8NA and the following lasers: 405, 488, 561, 640 nm. Images were analyzed using FIJI where fluorescent confocal images were occasionally subjected to equivalent brightness/contrast enhancement to improve image clarity.

We used Manders’ colocalization coefficients to describe the fraction of PANX1 colocalizing with a second target [49, 50]. Manders’ colocalization coefficient values range from 0.0 to 1.0 where values of 0.0 signify no pixel overlap and values of 1.0 denote identical spatial occupation between two signals [50]. We performed colocalization analysis in FIJI using the JACoP plugin with Coste’s automatic thresholding [51]. We report the standard error of the mean for Manders’ colocalization coefficients as indicated in the figure legends.

### Whole transcriptome analysis of PANX1 knockout using RNA sequencing

RNA was extracted using the PureLink™ RNA isolation kit (Cat # 12183018A, ThermoFisher) with on column DNase I digestion (Cat# 12185010, ThermoFisher) according to the manufacturers’ instructions. Purified RNA was quantified using a NanoDrop™ 2000 spectrophotometer (Cat# ND-2000, ThermoFisher), and stored at −80 °C until use. High quality RNA was identified by a ʎ260/280 of ≥ 2.0 and ʎ260/230 of ≥ 2.0.

Whole transcriptome analysis of gene expression differences in PANX1 knockout cells was carried out by RNA sequencing on a Illumina NovaSeq 6000 S4 PE100 (Genome Quebec). Paired end 100 bp reads were assessed for quality control using FastQC (version 0.11.9) [52]. Reads were aligned to the Human hg38 reference genome using RNA-Star (Galaxy Version 2.7.8a) with default settings [53] and transcripts were counted using featureCounts (Galaxy version 2.0.1) [54]. Differential expression of genes between control and *PANX1−/−* cells were based on a model using the negative binomial distribution with DeSeq2 (Galaxy Version 2.11.40.7), with a Benjamini-Hochberg adjusted *p*-value of less than 0.05 [55].

Identification of overrepresented groups of genes was carried out using GOseq (Galaxy Version 1.44.0) [56]. The three Gene Ontology (GO) categories were GO:MF (Molecular Function), GO:CC (Cellular Component), GO:BP (Biological Process). Distributions of the numbers of members of a category amongst the differentially expressed genes were determined by the Wallenius non-central hypergeometric distribution. *P*-values for over representation of the GO term in the differentially expressed genes were adjusted for multiple testing with the Benjamini-Hochberg procedure. GOseq was similarly applied for KEGG (Kyoto Encyclopedia of Genes and Genomes) pathway-based enrichment of differentially expressed genes.

### Quantitative reverse transcription PCR

High quality RNA was extracted from day 10 control and *PANX1−/−* organoids as described above. RNA was converted into complementary DNA (cDNA) using the High-Capacity cDNA Reverse Transcription Kit (Cat# 4368814, ThermoFisher) according to the manufacturer’s instructions. Typically, 500 ng of RNA were used per 20 µL cDNA reaction. The resulting cDNA was stored at −30 °C until use. Quantitative reverse transcription polymerase chain reaction (qPCR) was performed as previously described [12]. Primers shown in Table 5 were purchased from IDT (Integrated DNA Technologies, Coralville, IA, USA). Gene expression for each sample was normalized to *GAPDH*.

**Table 5:** Primer sets for qPCR.

Target	Forward primer (5′−3′)	Reverse primer (5′−3′)	Amplicon size (bp)
*GAPDH*	TGCTTTTAACTCTGGTAAAG	CACTTGATTTTGGAGGGATC	198
*GJA1*	GGTCTGAGTGCCTGAACTTGCCT	AGCCACACCTTCCCTCCAGCA	184
*PANX2*	Hs.PT.58.1348554		
*PANX3*	Hs.PT.58.4636086		

### SDS-PAGE & western blot

Cells were lysed with a solution comprising 50 mM Tris-HCl pH 8, 150 mM NaCl, 0.02% NaN_3_, 0.1% Triton™ X-100, 1 mM Na_3_VO_4_, 10 mM NaF, 2 µg/mL leupeptin, and 2 µg/mL aprotinin. Soluble proteins were separated using SDS-PAGE and transferred to a 0.45 µm nitrocellulose membrane (Cat# 1620115, Bio-Rad, Hercules, CA, USA). Primary antibodies (Table 4) were prepared in TBST (15.23 mM Tris HCl, 4.62 mM Tris Base, 150 mM NaCl, and 0.1% TWEEN® 20, adjusted to pH 7.6) + 3% BSA and incubated overnight at 4 °C. Secondary antibodies conjugated to HRP were prepared in TBST + 3% BSA and incubated for 1 h at room temperature. Proteins were visualized with Bio-Rad Clarity™ Western ECL Substrate (Cat# 1705061, Bio-Rad) using a ChemiDoc™ Imaging System (Cat# 12003153, Bio-Rad).

### Statistics

Statistical analyses were performed in GraphPad PRISM Version 9.4.1. Error bars depict ± standard error of the mean (SEM) when n ≥ 3 biological replicates (independent experiments) unless otherwise stated. Statistical significance for comparisons between 2 groups was determined by unpaired Student’s *t*-test. Statistical significance for comparisons between 3 or more groups was determined by Analysis of Variance (ANOVA) followed by a Tukey’s multiple comparisons test unless otherwise indicated. A *p* value less than 0.05 is considered statistically significant. *p < 0.05, **p < 0.01, ***p < 0.001, ****p < 0.0001.

### Supplementary information

Previous Article File

Full size Western blots

## Data Availability

The datasets generated during and/or analysed during the current study are available in the GEO repository (GSE249624).
