# Supplementary figures and images for: Apically localized PANX1 impacts neuroepithelial expansion in human cerebral organoids
Source: Cell Death Discov. 2024 Jan 11;10:22. doi: 10.1038/s41420-023-01774-7 (PMC10784521; doi:10.1038/s41420-023-01774-7)

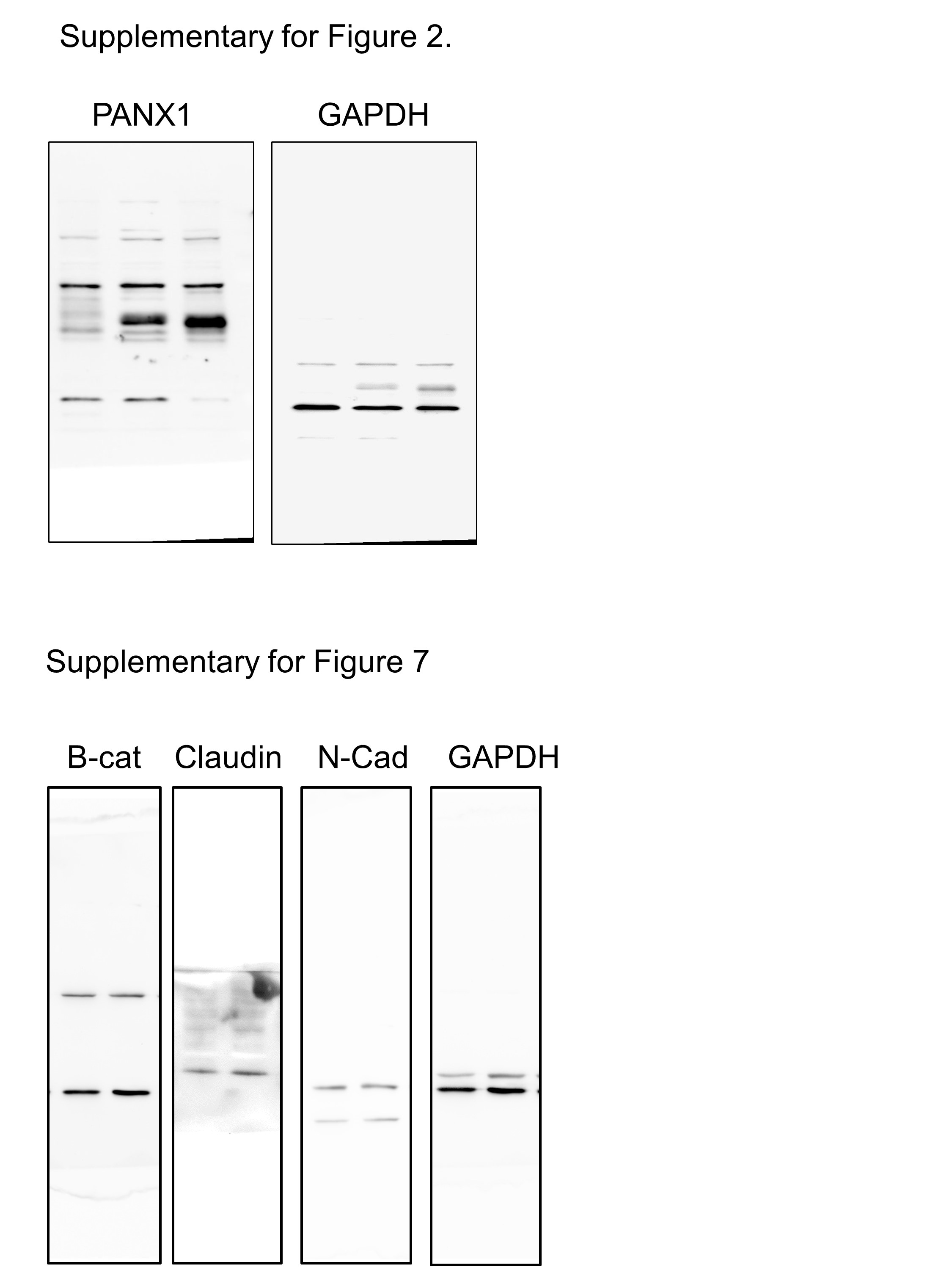

Supplement: Supplementary file 2 — Full size Western blots [file 41420_2023_1774_MOESM2_ESM.jpg]
